# Raised preoperative monocyte chemoattractant protein-1 as the independent predictor of delirium after cardiac surgery. A prospective cohort study

**DOI:** 10.1192/j.eurpsy.2021.676

**Published:** 2021-08-13

**Authors:** J. Kaźmierski, P. Miler, A. Pawlak, H. Jerczyńska, E. Frankowska, J. Woźniak, K. Woźniak, A. Brzezińska, M. Wilczyński

**Affiliations:** 1 Department Of Old Age Psychiatry And Psychotic Dosorders, Medical University of Lodz, Łódź, Poland; 2 Department Of Old Age Psychiatry And Psychotic Dosorders, Medical University of Lodz, Łódź (Łódź-Górna), Poland; 3 Department Of Affective And Psychotic Disorders, Central Clinical Hospital, Łódź, Poland; 4 Corelab Central Laboratory Of Medical University Of Lodz, Medical University of Lodz, Lodz, Poland; 5 Department Of Old Age Psychiatry And Psychotic Disorders, Central Clinical Hospital, Lodz, Poland; 6 Department Of Cardiac Surgery, Central Clinical Hospital, Lodz, Poland; 7 Department Of Cardiac Surgery, Medical University of Lodz, Lodz, Poland

**Keywords:** delirium, Cardiac surgery, Inflammation, Major depressive disorders

## Abstract

**Introduction:**

Delirium is a frequent and serious complication of cardiac surgery. However, the knowledge regarding pathogenesis of postoperative delirium is limited.

**Objectives:**

To investigate whether increased levels of monocyte chemoattractant protein-1 (MCP-1) and hyper-sensitive C-Reactive Protein (hsCRP) are associated with postoperative delirium in cardiac surgery patients.

**Methods:**

Patients were examined and screened for major depressive disorder (MDD) and cognitive impairment one day preoperatively, using the Mini International Neuropsychiatric Interview and The Mini-Mental State Examination Test. Blood samples were collected pre- and postoperatively for hsCRP and chemokine levels. Following surgical interventions, the Confusion Assessment Method for the Intensive Care Unit and the Memorial Delirium Assessment Scale with the cut-off score 10 were used to diagnose delirium.

**Results:**

Postoperative delirium screening was found positive in 34% (61 of 177) of patients. Both, pre- and postoperative hsCRP, and preoperative MCP-1 levels were associated with postoperative delirium in univariate comparisons; p=0.001; p=0.0004; p < 0.001, respectively. However, according to a multivariate stepwise logistic regression analysis only MCP-1 concentration raised before surgery was independently associated with postoperative delirium, and related to advancing age of participants (Spearman’s Rank Correlation 0.192; p=0.0103). According to ROC analysis, the most optimal cut-off for MCP-1 concentration in predicting the development of delirium was 371.81 ng/ml with sensitivity of 77.0% and specificity of 58.6%.

**Conclusions:**

The present study suggests that raised preoperative MCP-1 concentration is independently associated with delirium after cardiac surgery. Preoperative monitoring of pro-inflammatory markers combined with regular surveillance may be helpful in the prediction and early detection of postoperative delirium in this patient group.

